# Supplementary material for: Highly stable QLEDs with improved hole injection via quantum dot structure tailoring
Source: Nat Commun. 2018 Jul 4;9:2608. doi: 10.1038/s41467-018-04986-z (PMC6031613; doi:10.1038/s41467-018-04986-z)
Supplement: Supplementary file 1 — Supplementary Information [file 41467_2018_4986_MOESM1_ESM.pdf]

Highly stable QLEDs with improved hole injection via quantum dot structure tailoring

Cao et al.

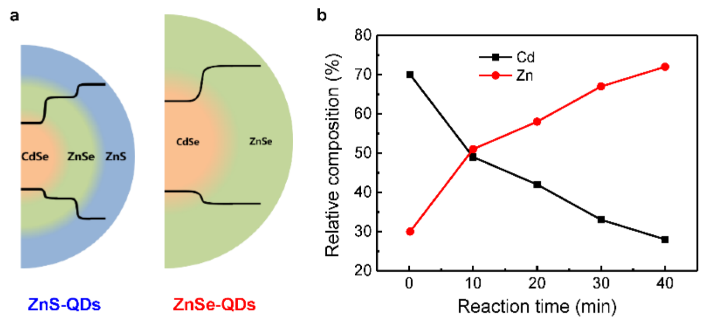

Supplementary Figure 1 | Composition of quantum dots. a, Schematics on detailed energy levels for a $CdSe/Cd_{1-x}Zn_xSe_{1-y}S_y/ZnS$ QD (ZnS-QD) and a $CdSe/Cd_{1-x}Zn_xSe/ZnSe$ QD (ZnSe-QD). **b**, Composition evolution of a typical ZnSe-QDs with the synthesis procedure described in the material synthesis section. The composition evolution of a ZnS-QDs is referred to Supplementary Ref. 1.

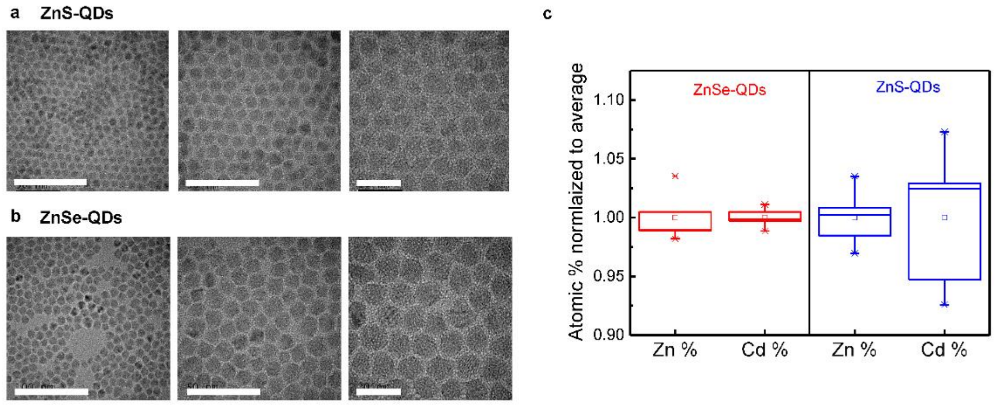

Supplementary Figure 2 | Characterization of quantum dots. a, TEM images of ZnS-QDs with different magnifications. **b**, TEM images of ZnSe-QDs with different magnifications. (The scale bars are 50 nm, 50 nm, and 20 nm from the left to the right). **c,** Normalized to average atomic percentage of Zn and Cd in ZnSe-QDs and ZnS-QDs measured by energy dispersive spectrometer (EDS), where the ZnSe-QDs exhibit a narrower composition distribution.

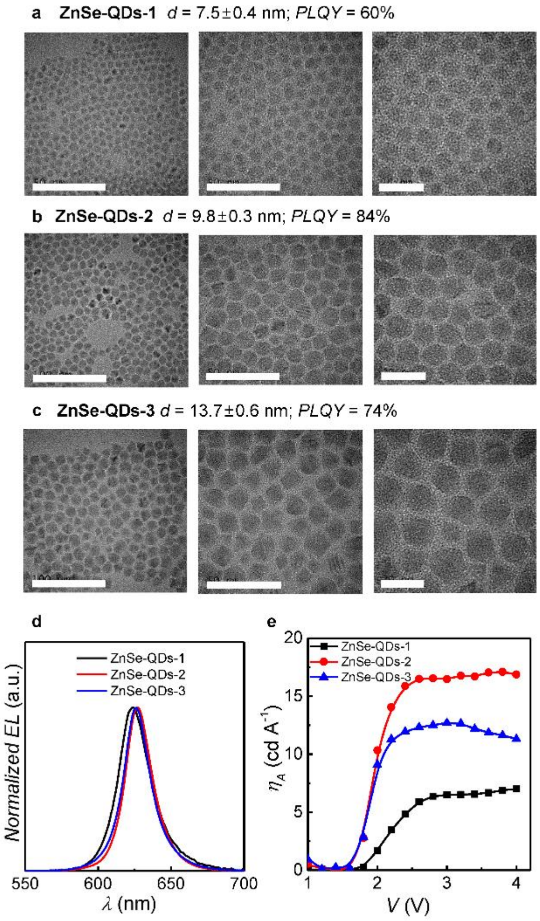

Supplementary Figure 3 | ZnSe-QDs optimization. a, b, c, TEM images of ZnSe-QDs with different magnifications. (The scale bars are 50 nm, 50 nm, and 20 nm from the left to the right.) These quantum dots were measured to be about 7.5 nm, 9.8 nm, and 13.7 nm in diameter, along with the *PLQY* of 60%, 84% and 74%, respectively. **d**, Electroluminescence spectra of the devices based on different ZnSe-QDs. **e**, Current efficiency (η_A) as a function of voltage for these devices.

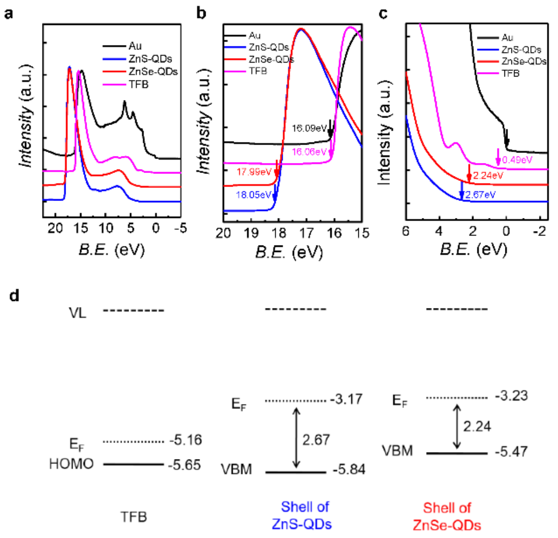

Supplementary Figure 4 | Energy level of the quantum dots. a, UPS spectra of ZnSe-QDs, ZnS-QDs, TFB and Au. **b, c,** Zoomed-in spectra of the secondary electron threshold (**b**) and valence band (**c**) regions. The work function of the Au reference sample is ~ 5.13 eV. **d,** The deduced energy level diagrams of the TFB layer and the shells of ZnS-QDs and ZnSe-QDs. UPS is surface sensitive with probing depth < 5 nm, thus, the shells of the QDs contribute mainly to the UPS signals here. The Fermi energy (E_F) of TFB, shell of ZnS-QDs and ZnSe-QDs are -5.16 eV, -3.17 eV and -3.23 eV, respectively. They are obtained by calculating the work function (ϕ) through $\phi = h\nu - E_{cut\text{-}off}$, where $E_{cut\text{-}off}$ is the measured secondary-cut-off energy and $h\nu$ is incident photon energy ($h\nu$=21.22 eV as HeI source is used here). The HOMO of TFB, VBM of ZnS-QDs and ZnSe-QDs shells are determined by the onset of photoelectron intensity with respect to E_F, which are -5.65 eV, -5.84 eV and -5.47 eV, respectively.[2]

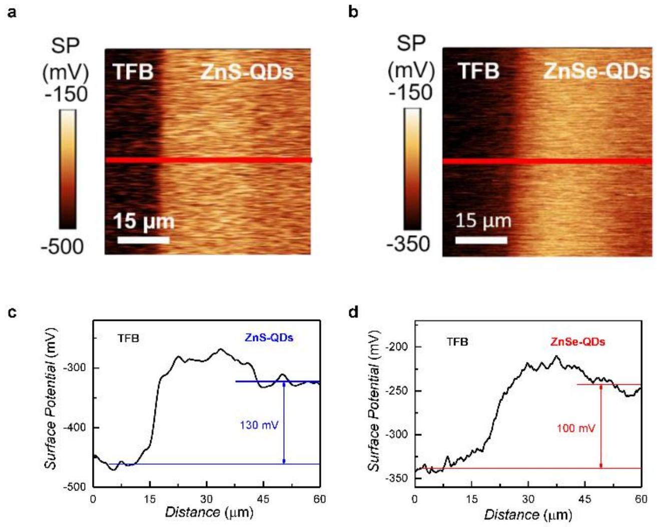

Supplementary Figure 5 | Electric dipole moment at TFB/QDs interface measured by SKPM. Surface potential images (**a, b**) and the extracted line profiles (**c, d**) of TFB surface partially covered with the ZnS-QDs (**a, c**) and ZnSe-QDs (**b, d**) layers.[3,4]

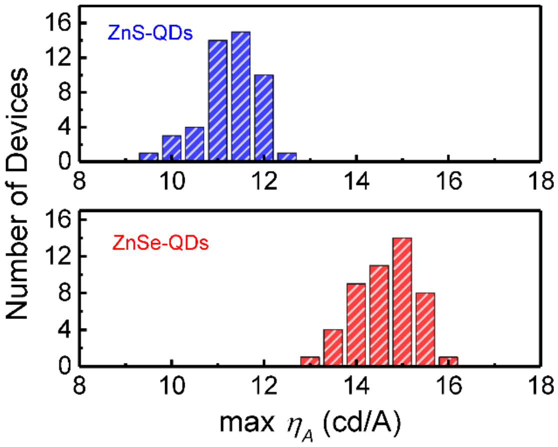

Supplementary Figure 6 | Statistics of device performance. Histograms of maximum current efficiency (max η_A) of 48 devices based on ZnS-QDs and 48 devices based on ZnSe-QDs.

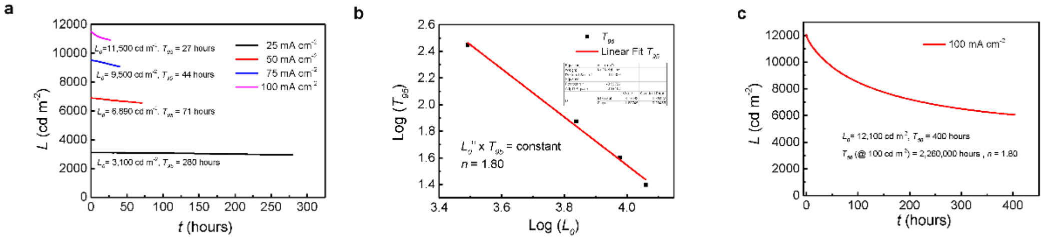

Supplementary Figure 7 | Device lifetime test. a, Stability data (luminance versus time) for a ZnSe-QDs device operated at different current density (25, 50, 75, 100 mA cm^{-2}).**b,** Extrapolation of accelerating factor (n) for the lifetime estimation by fitting the Log(T_{95})-Log(L_0) data points. Lifetime test was conducted under accelerated conditions to shorten the testing period, as commonly used in OLEDs. The initial luminance (L) and the measured time (t) for certain luminance degradation follows the empirical formula: $L^n \bullet t$ = constant. Here, n is defined as the accelerating factor, which can be obtained by fitting the values at multiple initial luminance. The lifetime at low luminance can therefore be extrapolated from the lifetime of the same device operated at high luminance following the equation. In our case, the accelerating factor is about 1.80. **c,** T_{50} lifetime of a ZnSe-QDs device operated at constant current density of 100 mA cm^{-2}.

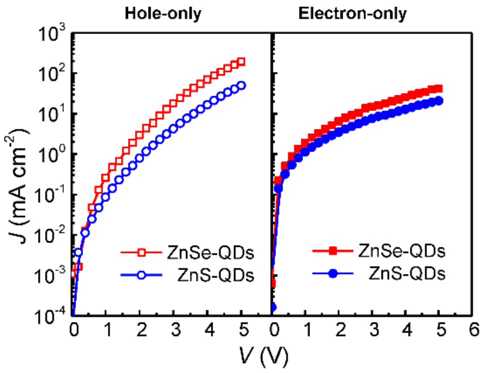

Supplementary Figure 8 | Single-carrier devices. Current density-voltage (*J*-*V*) characteristics of single-carrier devices based on ZnSe-QDs and ZnS-QDs. The hole-only devices have the structure of ITO (50 nm) /PEDOT:PSS (35 nm) /TFB (30 nm) /QDs (20 nm) /HAT-CN (5nm)/Ag (100 nm), and the electron-only devices have the structure of ITO (50 nm)/ZnO (40 nm) /QDs (20 nm)/ZnO (40 nm)/Al (100 nm).

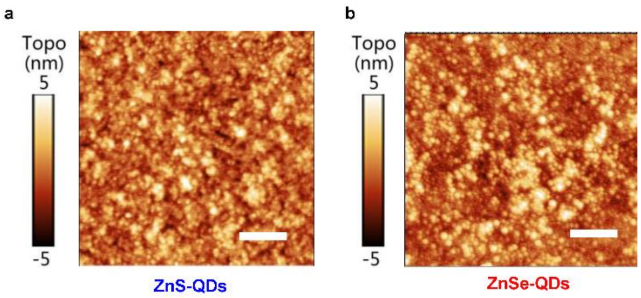

Supplementary Figure 9 | Surface morphology of QD films. Atomic force microscopy (AFM) topographies of of ITO/PEDOT:PSS/TFB/ZnS-QDs **(a)** and ITO/PEDOT:PSS/TFB/ZnSe-QDs **(b)** films. (The scale bars are 200 nm.) The root-mean-square (RMS) surface roughness are 1.42 nm and 1.85 nm for the ZnS-QDs and ZnSe-QDs films, respectively.

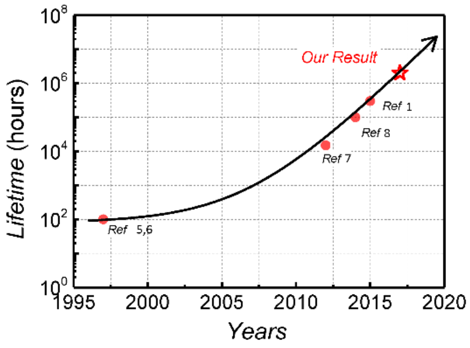

Supplementary Figure 10 | Comparison of the lifetime. The lifetime of our device is compared with other highly stable QLEDs.[1,5-8] The red, green and blue markers in the figure represent the red, green and blue emitting colors of QLEDs, respectively. All the data points shown in the figure are extrapolated to T_{50} lifetimes with the initial luminance of 100 cd m^{-2}. The early results of QLED lifetime are estimated to about 100 hours according to Supplementary Ref. 5 and 6. Supplementary Ref. 7 show a T_{50} lifetime at 500 cd m^{-2} of 600 hours, which are extrapolated to T_{50} lifetime at 100 cd m^{-2} of 15,000 hours, respectively, by assuming the accelerating factor of 2.0.

Supplementary References

1 Yang, Y. *et al.* High-efficiency light-emitting devices based on quantum dots with tailored nanostructures. *Nature Photon.* **9**, 259-266 (2015).

2 Kroupa, D. M. *et al.* Tuning colloidal quantum dot band edge positions through solution-phase surface chemistry modification. *Nature Commun.* **8** (2017).

3 Liang, X. *et al.* Colloidal indium-doped zinc oxide nanocrystals with tunable work function: rational synthesis and optoelectronic applications. *Chem. Mater.* **26**, 5169-5178 (2014).

4 Chen, Q. *et al.* Quantitative operando visualization of the energy band depth profile in solar cells. *Nature Commun.* **6** (2015).

5 Schlamp, M. C., Peng, X. & Alivisatos, A. P. Improved efficiencies in light emitting diodes made with CdSe(CdS) core/shell type nanocrystals and a semiconducting polymer. *J. Appl. Phys.* **82**, 5837-5842 (1997).

6 Mattoussi, H. *et al.* Electroluminescence from heterostructures of poly(phenylene vinylene) and inorganic CdSe nanocrystals. *J. Appl. Phys.* **83**, 7965-7974 (1998).

7 Qian, L., Zheng, Y., Xue, J. & Holloway, P. H. Stable and efficient quantum-dot light-emitting diodes based on solution-processed multilayer structures. *Nat Photonics* **5**, 543-548 (2011).

8 Dai, X. *et al.* Solution-processed, high-performance light-emitting diodes based on quantum dots. *Nature* **515**, 96-99 (2014).
